# What interventions do rural doctors think will increase recruitment in rural areas: a survey of 2778 health workers in Beijing

**DOI:** 10.1186/1478-4491-11-40

**Published:** 2013-08-21

**Authors:** Jinwen Wang, Jianglian Su, Huijuan Zuo, Mingyan Jia, Zhechun Zeng

**Affiliations:** 1 Beijing Institute of Heart, Lung and Blood Vessel Diseases, Capital Medical University Beijing Anzhen Hospital, No.2 Road Anzhen, Beijing, Chaoyang District, PR China; 2 Beijing Association of Medical Education, No.59 Road Beiwei, Beijing, Xicheng District, PR China

**Keywords:** Rural doctor, Recruitment, Human resources

## Abstract

**Background:**

A shortage of health professionals in rural areas is a major problem facing China, as more than 60% of the population lives in such areas. Strategies have been developed by the government to improve the recruitment of rural doctors. However, the inequitable distribution of doctors working in China has not improved significantly. The objective of this study was to explore the reasons for the poor recruitment and to propose possible strategies to improve the situation.

**Methods:**

Between September 2009 and November 2009 data were collected from 2778 rural doctors in Beijing, China. A quantitative survey was used to explore health workers’ perceptions as to what factors would have the greatest impact on recruitment and whether access to training had been effective in increasing their confidence, enhancing their interest in practicing medicine and increasing their commitment to recruitment.

**Results:**

Rural doctors were generally older than average in China. Of the 2778 participants, only 7.23% had obtained a license as a qualified doctor. For 53% of the rural doctors, the job was part-time work. The survey showed that rural doctors considered the training strategy to be inadequate. In general, the initiatives identified by rural doctors as being of most value in the recruitment of doctors were those targeting retirement pension and income.

**Conclusions:**

From the perspective of rural doctors, specific initiatives that promised a secure retirement pension and an increased income were considered most likely to assist in the recruitment of rural doctors in Beijing.

## Background

Health systems across the world face a shortage of skilled health workers in rural and remote areas, which hampers progress towards global health-care goals and contributes to inequalities in health outcomes [1-5]. In Beijing, the capital city of China, urban/rural disparities remain strong. Beijing suffers from a shortage of health workers, with only two doctors per 1000 inhabitants in 2006 in rural areas where more than 16.95 million people reside [6].

As a large agricultural country, China has a population of 1.34 billion with 674 million rural residents [7]. China established the Household Registration System in 1949, which divided the populace into rural residents and urban residents. The State Council released regulations on the official definitions of cities and towns. It stipulated that at least half the population of a rural township had to be in ‘agricultural’ work [8]. Beijing comprises 16 administrative sub-divisions, which are county-level units governed directly by the municipality. Of these, three districts are classified as urban districts as all the residents in the three districts are registered as being non-agricultural. More than half of the people living in the other 13 districts are registered as an agriculture population, and these administrative districts are divided into townships and villages.

There remains an urban–rural dual economic system in China. Whether measured in terms of income, literacy, or access to health services, a large gap is present between rural and urban areas. It was reported that the *per capita* income of an urban resident was 2.6 times higher than that of a rural resident in China. Typically, urban residents enjoy a better quality of education than rural residents. Furthermore, there is a skewed distribution of health-care services in favor of the urban sector [9-11]. According to the law on Licensed Doctors in China, ‘The Doctors’ refers to medical workers who have obtained licenses as qualified doctors and are registered and employed in medical services, disease prevention or health-care institutions. Anyone who has passed the examinations for the qualifications of a licensed doctor is certified as such. Whoever meets one of the following requirements may take the examinations for qualification as a licensed doctor: having graduated from a university faculty of medicineor having reached the level of a graduate from a faculty of medicine of a university or a polytechnic [12]. In urban areas, most doctors have completed a 5-year bachelor degree program in clinical medicine and obtained licenses as qualified doctors. They become either a family physician or a medical specialist. However, most rural doctors who serve the agricultural community are practicing without a license as a qualified doctor. After being registered at the county’s health bureau, rural doctors may practice at rural health-care clinics. Unlike their urban counterparts, rural doctors are not paid from public funds and are not eligible for social insurance. Their career prospects and training opportunities are poor [13].

Rural doctors have played a significant role in preventing people from becoming impoverished. In the mid 1950s, a collectivism-based rural health insurance system was established, called the Co-operative Medical System (CMS), which covered the cost of medical and obstetric care [14]. With the transition from a collective economy to a market system at the end of 1970s, the CMS collapsed, leaving around 90% of the rural population uninsured in the 1990s. Lack of health insurance increased the proportion of rural households living below the poverty line by 44% [15]. Rural doctors provided a series of health services to the local residents, including preventive services, maternal and child health services, and emergency medical aid. Despite a low level of service in terms of technique and medical instruments, the primary health-care services provided by rural doctors effectively reduced costs and provided timely treatment for the rural residents [16].

Researchers at the World Health Organization and other organizations have reported several specific recommendations to increase the availability of health workers in remote areas. These include financial incentive programs, training programs and improvement of working conditions [17-20]. In China, government strategies have been developed to decrease the inequity in health care between urban and rural areas, and these were expected to help improve the recruitment of rural doctors [15]. In 2003, the State Council and the Central Committee of the Communist Party of China initiated the policy of the New Cooperative Medical Scheme (NCMS) [21]. This is a health insurance scheme for rural residents, which is designed to reduce the financial burden of illness on the rural population. In addition, a fixed special government allowance for rural doctors has been introduced in many regions. Since 2006, the Chinese government has initiated large-scale training programs aimed at improving the knowledge and skills of rural doctors.

The coverage of the new strategies had expanded to 2451 counties by the end of 2007, accounting for 86% of all rural counties in China. A recent study found that the implementation of the NCMS significantly increased the use of preventive care [22]. There was an improvement in the working environment of rural doctors in Beijing. Beijing Municipal People’s Government provides an 800-yuan ($130) monthly allowance to each rural doctor. In 2006, a medical training program was initiated by the Beijing Municipal Health Bureau to improve the basic clinical skills and recruitment of rural doctors. A 6 million yuan ($1.0 million) special fund was spent on the training in Beijing. Ten thousand rural doctors participated in the training from 2006 to 2009. However, the impact of the strategies on recruitment of rural doctors is still limited. Although the report released by the Chinese Ministry of Health showed the number of rural doctors increased by 6.5% between 2003 and 2008, from 868000 to 938000, the overall level of human resources suggests a critical shortage in rural areas in China. China’s urban doctor-patient ratio was 2.8 doctors per 1000 residents in 2011. In rural areas, the ratio was 0.95 doctors per 1000 residents [15].

The problem of health workforce adequacy is operationally and conceptually complex. Intensive and sustained effort is needed to rectify the multidimensional nature of the recruitment of rural doctors. However, there has been very limited research to determine the major requirements of rural doctors in China. The objective of this study was to identify interventions proposed by the government that would lead to improved recruitment from the perspective of rural doctors in Beijing.

## Methods

Between September 2009 and November 2009 data were collected from 2778 rural doctors in village clinics in the townships and villages of the 13 rural districts of Beijing Municipality. Health care is provided through a three-tiered system in Beijing. In rural areas the first tier is made up of 10000 rural doctors working out of village clinics. At the next level are the 184 community health centers, which function primarily as outpatient clinics. The third and final tier is the 13 rural district hospitals, which are staffed by senior doctors. By referring to government records, we listed all the village clinics associated with the 184 community health centers. For each community health center, we randomly selected four village clinics. The resulting sample included 736 village clinics and 2801 individual doctors. Participants were provided with invitations to participate by direct contact with the hospital in-charge officer. Finally, 2778 rural doctors agreed to participate and completed the survey in the local secondary hospitals. The overall response rate was 99.2%. All information was collected with the help of the Beijing Association of Medical Education and the township division of the Beijing Municipal Health Bureau.

The development of the questionnaire was based on a review of formally published literature and policy documents on staff recruitment in rural areas. The questionnaire covered respondents’ backgrounds and personal demographic characteristics. Possible interventions that were relevant to recruitment were listed in the questionnaire. Respondents were asked to assign a rank to indicate which intervention would help most to attract more rural doctors to village clinics (1 = most important to 6 = least important).

All participants accepted the medical training organized by the Beijing Municipal Health Bureau from 2006 to 2009. Data were collected about the impact of the training on rural doctors with the question: ‘Can training help to attract more doctors to work in the rural area?’ We used a 5-point Likert scale to identify whether respondents strongly agreed, agreed, were neutral, disagreed or strongly disagreed with the statements.

The study was approved by the Beijing Municipal Health Bureau. Analysis was performed using SPSS (SPSS Inc., Chicago, IL, USA) to calculate item means and to determine the 95% confidence intervals.

## Results

Table 1 summarizes the characteristics of the responders. Of the 2778 participants, 1423 (51.2%) were men and 1355 (48.8%) women. The mean age of the respondents was 50 years (standard deviation, 8.02; range, 24–75 years). In total, 76.5% had been working in village clinics for more than 20 years, and only 7.23% had obtained a license as a qualified doctor. Only 85 health workers became rural doctors in Beijing over the past 10 years and accounted for 3.1% of all the rural doctors. Half of the rural doctors (50.3%) worked part-time. Qualified rural doctors compared with non-qualified rural doctors were younger, better educated and 1.5% had a university degree. A higher proportion of rural doctors who were qualified tended to have higher household income, with 45.2% exceeding 2000 yuan ($242) a month compared with 25.5% of non-qualified rural doctors and were more likely to work full-time.

**Table 1:** Demographics of participants

**Characteristics**	**All (no. = 2778)**	**Non-qualified rural doctors (no. = 2577)**	**Qualified rural doctors (no. = 201)**
Gender			
Female no. (%)	1355 (48.8%)	1252 (48.6%)	103 (51.2%)
Male no. (%)	1423 (51.2%)	1325 (51.4%)	98 (48.7%)
Age			
Mean age	50.01 ± 8.02	48.62 ± 9.49	50.16 ± 8.00
Range (year)	24–75	28–62	24–75
<40 year no. (%)	481 (17.3%)	433 (16.8%)	48 (23.9%)
40–60 year no. (%)	2069 (74.5%)	1930 (74.9%)	139 (69.2%)
>60 year no. (%)	228 (8.2%)	214 (8.3%)	14 (7.0%)
Years of medical service			
Mean length	27.90 ± 9.45	28.10 ± 9.43	25.80 ± 9.10
<10 no. (%)	85 (3.1%)	78 (3.0%)	7 (3.5%)
10–19 no. (%)	568 (20.4%)	516 (20.0%)	52 (25.9%)
20–29 no. (%)	578 (20.8%)	529 (20.5%)	49 (24.4%)
>30 no. (%)	1547 (55.7%)	1454 (55.6%)	93 (45.8%)
Education			
University no. (%)	26 (0.9%)	23 (0.9%)	3 (1.5%)
High school & Junior college no. (%)	299 (10.8%)	250 (9.7%)	49 (24.4%)
Primary & secondary school no. (%)	2453 (88.3%)	2304 (89.4%)	149 (74.1%)
Have a part time job	1472 (50.3%)	1378 (53.4%)	94 (40.8%)
Per capita monthly household income			
<2000 yuan no. (%)	2030 (73.1%)	1920 (74.5%)	110 (54.7%)
2000–4000 yuan no. (%)	622 (22.4%)	557 (21.6%)	65 (32.3%)
>4000 yuan no. (%)	126 (4.5%)	100 (3.9%)	26 (12.9%)

The interventions and their rank, which indicated the relative importance of each item with respect to the recruitment of rural doctors, are listed in Table 2. The most notable finding was that the retirement pension and income were the most important issues for rural doctors, whereas ‘the chance of launching a successful career’ was ranked as the least important item.

**Table 2:** Mean ranks for the importance of intervention items, with the lowest scores having the greatest impact

**Intervention item**	**Mean rank (mean ± 95% CI)**
1. Retirement pension offered by government	1 (1.67 ± 0.09)
2. Increase income	2 (2.98 ± 0.10)
3. More training	3 (3.89 ± 0.11)
4. Reasonable workload and improved after-hours	4 (4.19 ± 0.09)
5. Improved working environment	5 (5.54 ± 0.08)
6. A better chance of launching successful careers	6 (5.87 ± 0.10)

*CI* confidence interval.

The results according to age are displayed in Table 3. Analysis by age found agreement on the two most important items: ‘retirement pension offered by the government’ and ‘increased income’. However, there were different views between different age groups on the least important intervention to attract rural doctors into rural areas. More rural doctors <40-years-old felt that launching a successful career was more important than older rural doctors.

**Table 3:** Mean ranks for the importance of intervention items by age category

**Intervention item**	**Mean rank (mean ± 95% CI)**		
	**>60 years old**	**40–60 years old**	**<40 years old**
1. Retirement pension offered by government	1 (1.64 ± 0.09)	1 (1.70 ± 0.09)	1 (1.72 ± 0.09)
2. Increase income	2 (2.87 ± 0.10)	2 (2.99 ± 0.10)	2 (3.10 ± 0.10)
3. More training	3 (3.89 ± 0.11)	3 (3.60 ± 0.11)	3 (3.57 ± 0.11)
4. Reasonable workload and improved after-hours	4 (4.10 ± 0.09)	4 (4.12 ± 0.09)	4 (4.26 ± 0.09)
5. Improved working environment	5 (5.24 ± 0.08)	5 (5.33 ± 0.08)	5 (5.76 ± 0.08)
6. The more chance of launching successful careers	6 (5.92 ± 0.10)	6 (5.89 ± 0.10)	6 (5.56 ± 0.09)

*CI* confidence interval.

Table 4 indicates the changes resulting from training and how much these changes resolved the problem of recruitment. The results indicated that participants generally shared the perception that training had specifically contributed to improvement of two out of six factors, namely ‘improved clinical knowledge’ and ‘enhanced clinical skills’. The statements ‘I feel more confident as a rural doctor after training’ and ‘training can help to attract more young doctors to work in rural areas’ were considered neutral overall. The participants generally disagreed that ‘training is valuable to me in my future career and ‘training enhanced my interest in medical practice’.

**Table 4:** Response to statements on training

**Statement**	**5 = Strongly disagree % (no.)**	**4 = Disagree % (no.)**	**3 = Neutral % (no.)**	**2 = Agree % (no.)**	**1 = Strongly agree % (no.)**
1. The training has improved my clinical knowledge	0%(0)	12%(333)	14%(389)	48%(1333)	26%(722)
2. The training has enhanced my clinical skills	0%(0)	18%(500)	11%(306)	47%(1306)	24%(667)
3. The training is valuable to me in my future career	28%(778)	49%(1361)	16%(444)	7%(194)	0%(0)
4. The training enhanced my interest in medical practice	34%(945)	41%(1139)	12%(333)	11%(306)	1%(28)
5. I feel more confident as a rural doctor after training	6%(167)	31%(861)	24%(667)	34%(945)	5%(139)
6. The training can help to attract more young doctors to work in rural areas	7%(194)	30%(833)	31%(861)	29%(806)	3%(83)

## Discussion

A shortage of health personnel is one of the constraints in meeting population health needs, and the present study found that Beijing faced problems of recruitment of doctors in rural areas in recent years. There has been some debate in the literature on the recruitment of rural doctors in developing countries. Studies have shown that recruitment is influenced by both financial and non-financial incentives. Improved salary, improved working conditions and access to training are motivating factors for health workers [23-25]. In our study, the initiative identified by rural doctors as being of most value in the recruitment of doctors was their retirement pension.

In the course of economic transition, China adopted an urban-priority reform path in establishing its old-age social security system. The insurance pilot project has not yet been launched nationwide. China’s pension system now has a high coverage rate for urban workers, but a low coverage rate for rural farmers. Shijiaqi et al. reported that 97% of village doctors had no access to basic endowment insurance [26]. It is not surprising, therefore, that our study discovered that access to a retirement pension was an important incentive for health workers when considering rural practice.

The findings suggest that income also has an influence on a rural doctor’s choice of practice location. The average income of urban doctors is about 3000 yuan ($500) per month [27]. In the present study, although qualified rural doctors tended to have higher household incomes compared with non-qualified doctors, 87% of qualified rural doctors did not reach the household income level of 4000 yuan per month. Their medical practice income is not enough to maintain a basic standard of living. It is also not surprising that some qualified rural doctors had agricultural jobs alongside practicing medicine.

A number of factors contribute to the poor welfare of rural doctors in China [28]. The Household Registration System prohibited the free migration of farmers to prevent the impact on the urban welfare system. In the 1950s, ‘barefoot doctors’ who were semi-medical trained farmers provided most of the primary care services in rural areas. The income of the barefoot doctors was calculated as if they performed agricultural work, and they were paid roughly half of what a properly trained doctor was paid [16]. In the 1980s, the term ‘barefoot doctor’ was banned by the Ministry of Health, and some became rural doctors. However, under China’s unique Household Registration System, rural doctors are still classified as farmers and do not enjoy urban welfare benefits such as heavily subsidized housing, medical care and pensions [27]. The Household Registration System is perceived as the primary barrier to population mobility in China even today. Government should accelerate reform of the Household Registration System and establish the principle of equity. With regard to labor insurance and other welfare benefits, rural doctors should enjoy the same benefits as enjoyed by their urban counterparts.

Although other studies showed that appropriate training would strengthen the self-esteem of doctors and may improve recruitment, this study did not support this. White et al. reported that medical training was extremely effective in increasing practitioners’ confidence in practicing in rural and remote communities, reducing professional isolation and increasing commitment to remain in rural practice [29]. In our study, some rural doctors reported that the current training system had a positive effect on their enhancement of medical skills. However, nearly two-thirds of the sample (61%) did not consider the training to be important for encouraging young doctors to work as rural doctors. The disappointing result suggests that the problem of recruitment may be more complex than in other countries. The urban–rural economic gap has continued to widen in recent years [30]. Although training was reported to be an effective form of incentive for recruitment, it may not be enough to attract young doctors to rural areas in China.

The results suggest that access to the social pension system and significant salary increases for rural doctors would have some impact on hard-to-staff areas. The Government of China is already pursuing a number of policy measures to improve the basic pension system and to eliminate the income gap between rural and urban areas [31]. However, these are seen as having had limited effectiveness to date. Addressing the differentials is likely to require increasing the country’s social security funds through multiple channels, as well as strengthening oversight and supervision to ensure the implementation of national policies.

The present study has some limitations. First, profiles of doctors and environmental factors are likely to vary geographically, and, hence, the context in Beijing may not be representative of all rural areas in China. Second, the present study did not explore the reasons why qualified rural doctors chose to work in the village clinics. Further research on recruitment of rural doctors is needed to explore these questions.

## Conclusions

Economic factors have largely been the focus of recruitment initiatives in Beijing. From the perspective of rural doctors, specific initiatives that promise secure retirement pension, and an increase in income are considered those most likely to assist in the recruitment of rural doctors in Beijing.

## Competing interests

The authors declare that they have no competing interests.

## Authors’ contributions

The work presented here was carried out in collaboration between all authors. JW, ZZ and MJ defined the research theme. JW and ZZ designed methods. JW, JS, ZZ and HZ designed the questionnaires, carried out the data collection, analyzed the data and interpreted the results. JW wrote the manuscript. MJ provide administrative support. All authors read and approved the final manuscript.
